# Multiaxial vibration dataset obtained on a triaxial electrodynamic shaker rig using multiple control strategies

**DOI:** 10.1016/j.dib.2026.113008

**Published:** 2026-06-22

**Authors:** Christophe Gautrelet, Leila Khalij, Carole Dovillaire, Olivier Bareille

**Affiliations:** a INSA Rouen Normandie, Normandie Univ, LMN UR 3828, F-76000, Rouen, France; b Spectral Dynamics SAS, 38500, Voiron, France

**Keywords:** Experimental practices, Triaxial vibration testing, Control strategies, Sweep-sine tests, Random tests, Dynamic responses

## Abstract

This article presents an experimental vibration dataset obtained on a triaxial electrodynamic shaker rig, equipped with a magnesium cube fixture instrumented with five triaxial and three single-axis accelerometers. Eleven sweep-sine and random vibration tests were conducted over the 5–2000 Hz frequency range using four closed-loop control strategies and acquisition settings corresponding to 1600 frequency lines (1.25 Hz frequency resolution). The dataset includes raw, Frequency Response Functions (FRFs), Power Spectral Densities (PSDs), and coherence matrices acquired using the Jaguar MIMO control system. The measurements highlight the strong influence of fixture resonances and sensor location on spatial acceleration variability and control stability, particularly above 1500 Hz where low-amplitude compression was required. The dataset can support benchmarking of multiaxial control strategies, assessment of spatial variability, and validation of numerical models for structural dynamics and vibration qualification.

Specifications Table

**Table:** 

Subject	Engineering & Materials science
Specific subject area	Dynamics, vibration testing
Type of data	Raw and processed data; Tables; Figures
Data collection	Data were collected on a triaxial electrodynamic shaker rig (TIRA/Kokusai/Spectral Dynamics). Accelerations were measured using five triaxial and three monoaxial accelerometers. Signal generation and closed-loop control were performed using Spectral Dynamics' Jaguar hardware and software. Five triaxial accelerometers were mounted on the top surface of the cube fixture and three single-axis accelerometers were mounted on its side surfaces. Sweep-sine tests were conducted at a sweep rate of 1 octave per minute at a level of 1.5 g, while random vibration tests were performed at 1 *g*-RMS for five minutes. The frequency resolution was fixed at 1.25 Hz, corresponding to 1600 frequency lines over the analysed frequency range. The dataset includes raw spectra and data post-processed using MATLAB.
Data source location	Mechanical Laboratory of Normandie (LMN), INSA Rouen Normandie, Avenue de l’université, 76,800 Saint Etienne du Rouvray, France
Data accessibility	Repository name: Mendeley Data identification number: doi: 10.17632/d2tfzht9sf.1 Direct URL to data: https://data.mendeley.com/drafts/d2tfzht9sf.1 Instructions for accessing these data: Open the Mendeley Data record and download the dataset archive. The repository contains raw data, processed data (FRFs/PSDs), and MATLAB figure files.
Related research article	*None*

## 1 Value of the Data

• These multiaxial vibration measurements provide a benchmark dataset for evaluating several multi-point control strategies, including single triaxial accelerometer control, distributed triaxial accelerometer control, distributed monoaxial control, and multi-point rectangular-control configuration, under both sweep-sine and random vibration tests.
• The dataset is intended for the vibration-testing and structural-dynamics communities, particularly for researchers and engineers interested in the behaviour of triaxial electrodynamic shaker rigs. The provided FRFs and PSDs dataset allow evaluation of control performance in terms of stability, spatial variability, accuracy, and cross-axis coupling effects, which are critical aspects of vibration qualification and multiaxial testing.
• The dataset covers a wide frequency range (5–2000 Hz), providing diverse operating conditions for further studies on sensor-placement optimisation, controller parameter tuning, and response characterisation under multiaxial loading. During sweep-sine testing, the control performance was degraded in the upper frequency range. Consequently, low-amplitude compression was intentionally applied above 1500 Hz to maintain control-loop stability and to prevent excessive actuator drive in this band.
• The FRF measurements can also support identification of modal characteristics specific to the shaker-fixture assembly, thereby improving the correlation between experimental observations and numerical models.

## 2 Background

Multiaxial vibration environments can significantly influence the response and durability of engineered systems. Conventional qualification is commonly performed on single-axis shakers, where multiaxial loading is approximated by sequential excitation along each orthogonal axis, as in standards such as MIL-STD-810 G [1]. However, real structures experience simultaneous vibrations in multiple directions, and cross-axis interactions may significantly alter the structural response [2].

To overcome these limitations, multiaxial vibration shaker rigs have emerged in recent years. Notably, the LMN triaxial electrodynamic shaker rig was acquired through European funding via the Normandy Region and is operated to reproduce more realistic loading conditions along three orthogonal axes. The triaxial shaker rig can be used for vibration testing of structures or assemblies made of various materials, provided that their dimensions and mass remain compatible with the shaker-table capacity and targeted acceleration levels. In practical applications, fixture interfaces are generally specifically designed according to the tested structure and the mechanical conditions to be investigated. Despite this capability, cross-axis coupling effects on measured responses and control outcomes remain insufficiently documented [3].

The system is equipped with a magnesium cube fixture featuring an internal design optimised to position the centre of gravity. Internal design details of the fixture are proprietary information from the manufacturer and are therefore not publicly available. However, the fixture exhibits multiple structural resonances within the 5–2000 Hz frequency range, leading to non-uniform acceleration distributions depending on the sensor location and excitation conditions.

Consequently, the measured acceleration is not spatially uniform and control performance can depend strongly on sensor location. Recent studies have highlighted the importance of advanced vibration-control strategies, sensor placement optimisation, and cross-axis coupling management in multiaxial testing and structural monitoring applications [[4], [5], [6], [7], [8], [9]].

These aspects become particularly critical when multipoint control configurations are implemented over wide frequency ranges. In this context, the present dataset enables a systematic comparison of four acceleration-control strategies on a triaxial electrodynamic shaker rig, with particular emphasis on the influence of sensor location on control performance [10].

The dataset was designed to support the comparison of several control configurations and sensor placement under sweep-sine and random excitations. It includes raw recordings and processed quantities (PSDs and FRFs) and can support practical decisions regarding test bandwidth definition and frequency-band selection for qualification and fatigue testing.

## 3 Data Description

The dataset consists of acceleration measurements recorded during a triaxial vibration test campaign aimed at assessing how different control strategies influence the dynamic response of the shaker rig.

Data are organised into the following categories:

● Sweep-sine test data (Raw data/3D sweep-sine).
● Random vibration test data (Raw data/3D Random).
● Processed data (FRFs and PSDs).
● MATLAB Figures.

Each data folder contains subdirectories corresponding to the four control strategies:

● (S1) single-point control using one triaxial accelerometer.
● (S2) control using three single-axis accelerometers (one per axis).
● (S3) distributed-control configuration using three triaxial accelerometers.
● (S4) multi-point rectangular-control configuration using three accelerometers [11].

The dataset is provided as MATLAB ASCII files generated with the Jaguar Data Conversion utility. The file formats correspond to:

● Raw sweep-sine spectra are provided as ASCII files (file extension: .frq). Each row contains the frequency (Hz) followed by amplitude/phase pairs for the 18 acquisition channels. Amplitudes are expressed in g and phases in degrees.
● FRFs are provided as ASCII files (file extension: .frf), where each row contains the frequency (Hz) followed by magnitude/phase pairs (the number of columns depends on the number of exported FRFs); FRF magnitudes are expressed in g/g (dimensionless) and phases in degrees.
● Random results are provided as ASCII files: PSD files contain the frequency (Hz) and PSD values for channels 1–18 in g²/Hz (19 columns).
● Coherence matrices are provided as ASCII files (file extension: .coh): only the diagonal and upper-triangular terms are stored in the converted files.

Despite the compression strategy applied during sweep-sine testing, the multi-point rectangular-control configuration was not always successfully. The unsuccessful rectangular-control attempts were not retained in the released dataset because the control loop became unstable at an early stage of the tests and the acquisitions were interrupted before stable and exploitable measurements could be obtained.

### 3.1 Sweep-sine acceleration dataset

**Fig. 1:**
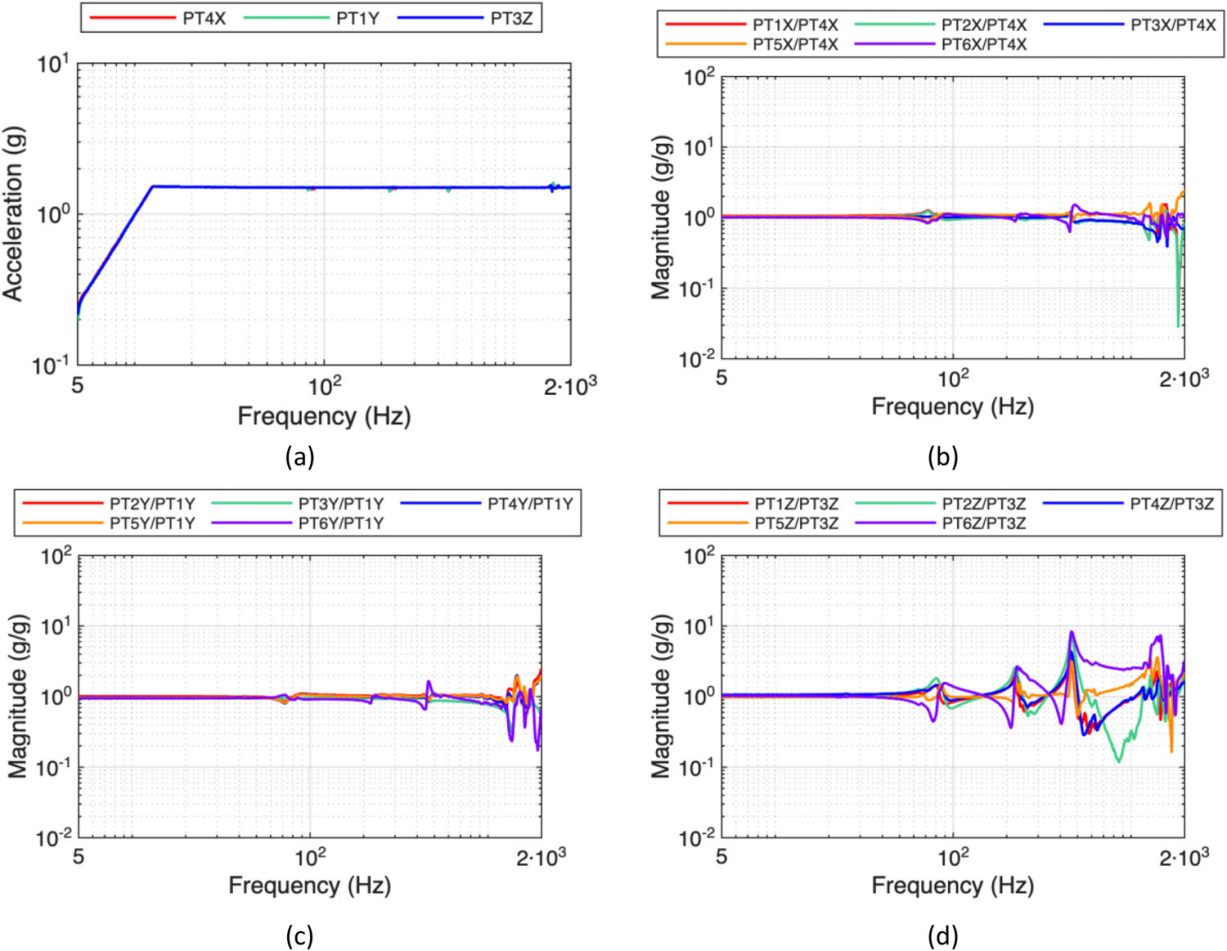
(a) Raw acceleration spectra and (b-d) corresponding FRFs obtained for the distributed-control configuration using PT4X, PT1Y and PT3Z as control sensors (S3, ID5).

Representative examples of sweep-sine measurements are presented for the distributed-control configuration using three triaxial accelerometers, one per axis (S3, ID5): PT4 for X (channel 10), PT1 for Y (channel 2), and PT3 for Z (channel 9). The corresponding acceleration spectra and FRFs are shown in Fig. 1.

Additional FRF and PSD comparisons for the other control configurations are provided in the supplementary material and remain fully accessible through the Mendeley repository.

### 3.2 Random acceleration dataset

**Fig. 2:**
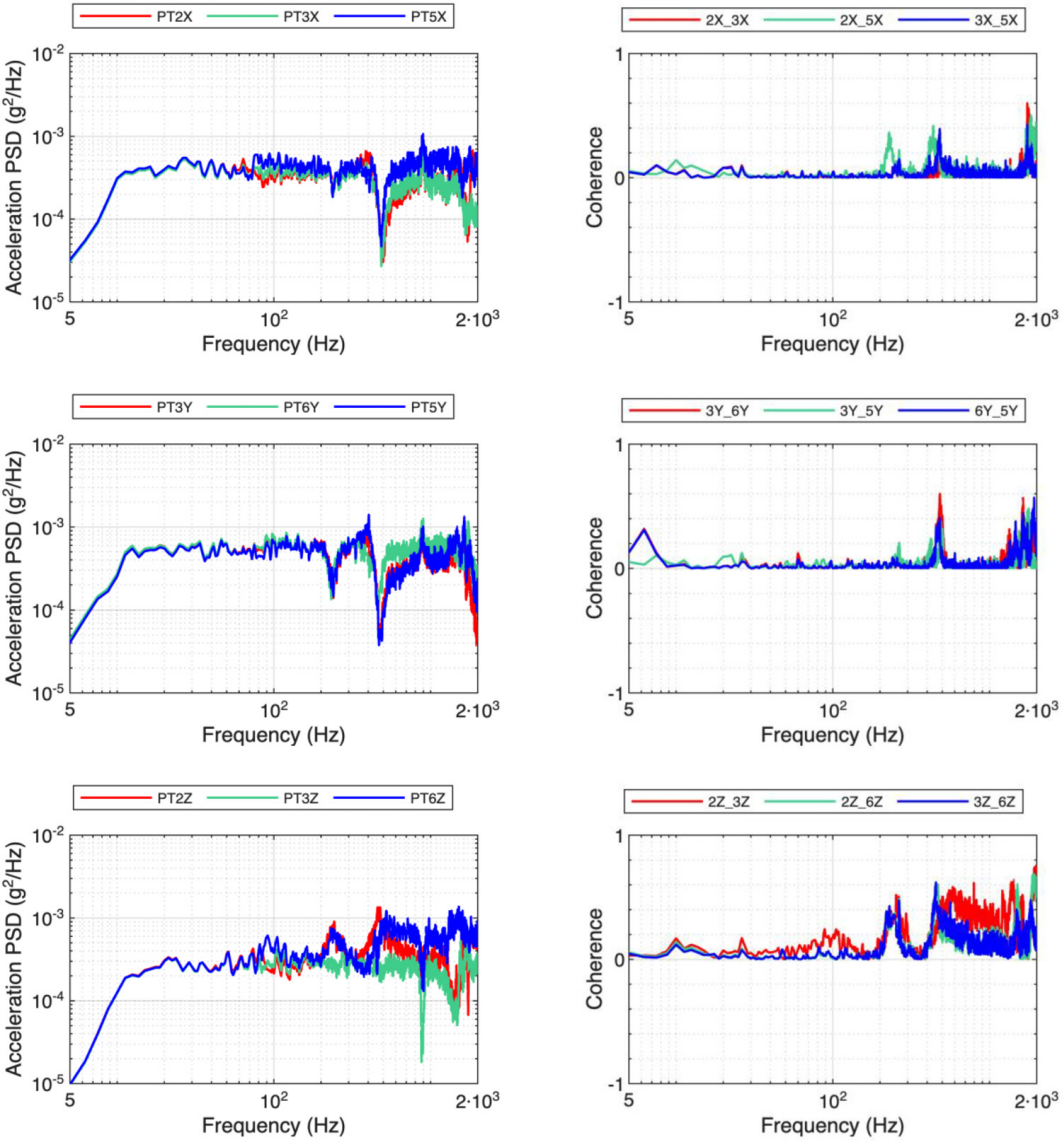
Raw PSDs (left) and associated cross-coherences (right) recorded for the multi-point rectangular-control configuration using three accelerometers per axis (S4, ID11).

**Fig. 3:**
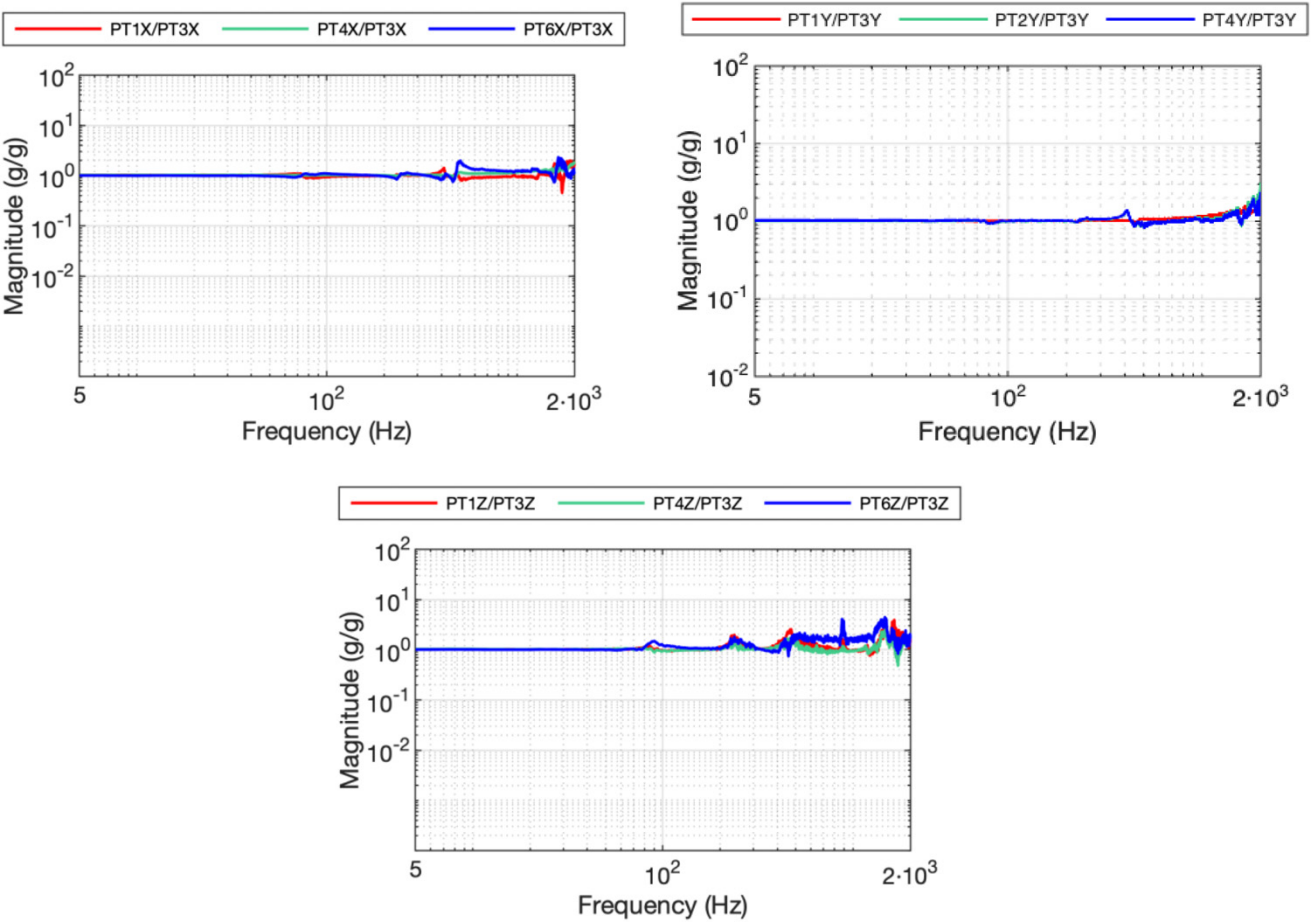
Corresponding FRFs for the multi-point rectangular-control configuration (S4, ID11). The centre of the cube fixture (PT3) was used as the reference location for response comparison.

Representative examples of random-vibration measurements are presented for the multi-point rectangular-control configuration using three accelerometers per axis (S4, ID11): PT2, PT3 and PT5 for X; PT3, PT5 and PT6 for Y; and PT2, PT3 and PT6 for Z. The corresponding PSDs, cross-coherences, and FRFs are shown in Fig. 2, Fig. 3.

Additional comparisons for the other control configurations are provided in the supplementary material and remain fully accessible through the Mendeley repository.

### 3.3 How the data can be reused

The dataset can be reused for:

● Benchmarking control strategies for multiaxial vibration shaker rigs.
● Studying cross-axis coupling and its impact on closed-loop behaviour.
● Validating numerical models using FRFs over a wide frequency range.
● Developing vibration-analysis algorithms (e.g., modal identification, anomaly detection).
● Supporting educational activities related to advanced vibration testing methods.

## 4 Experimental Design, Materials and Methods

### 4.1 Experimental setup

The experimental setup consisted of a triaxial air-cooled electrodynamic shaker system composed of three orthogonally mounted shakers (TIRA/Kokusai/Spectral Dynamics), controlled using a Spectral Dynamics Jaguar ACP (Acquisition & Control Peripheral) controller and acquisition unit (Fig. 5).

**Fig. 4:**
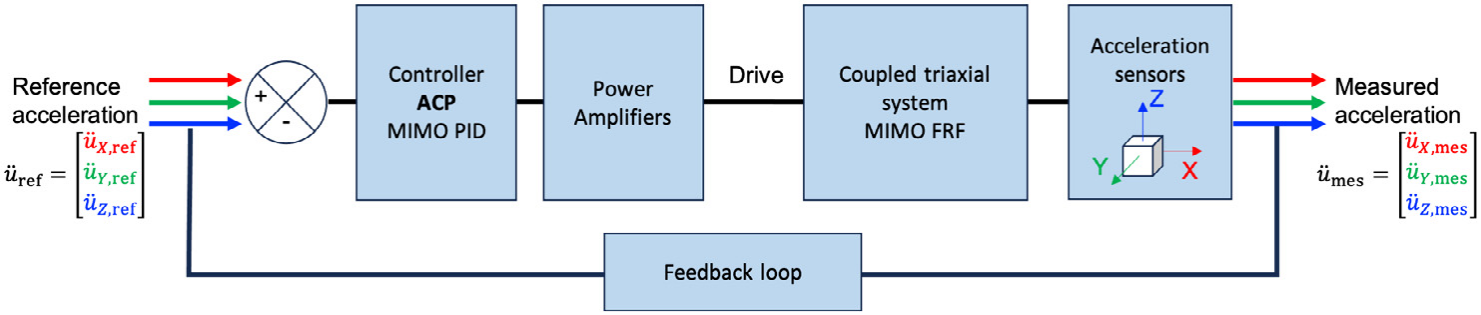
Closed-loop control architecture of the triaxial shaker rig.

Fig. 4 illustrates the closed-loop vibration-control architecture implemented using the Jaguar hardware, including the generation of the reference excitation spectra, the Jaguar ACP controller, the power amplifiers, the triaxial shaker rig, and the accelerometer feedback loop used for real-time control and response acquisition of one, two, or three shakers simultaneously.

**Fig. 5:**
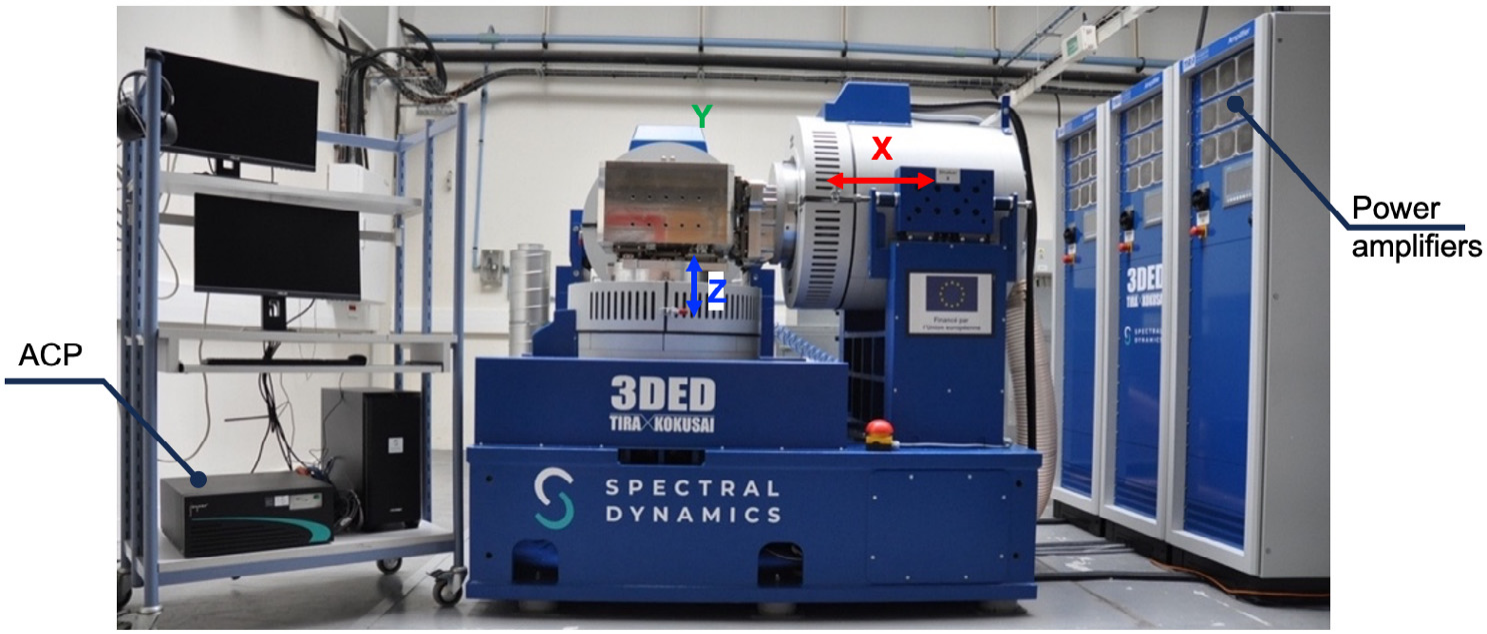
The triaxial electrodynamic shaker rig.

**Fig. 6:**
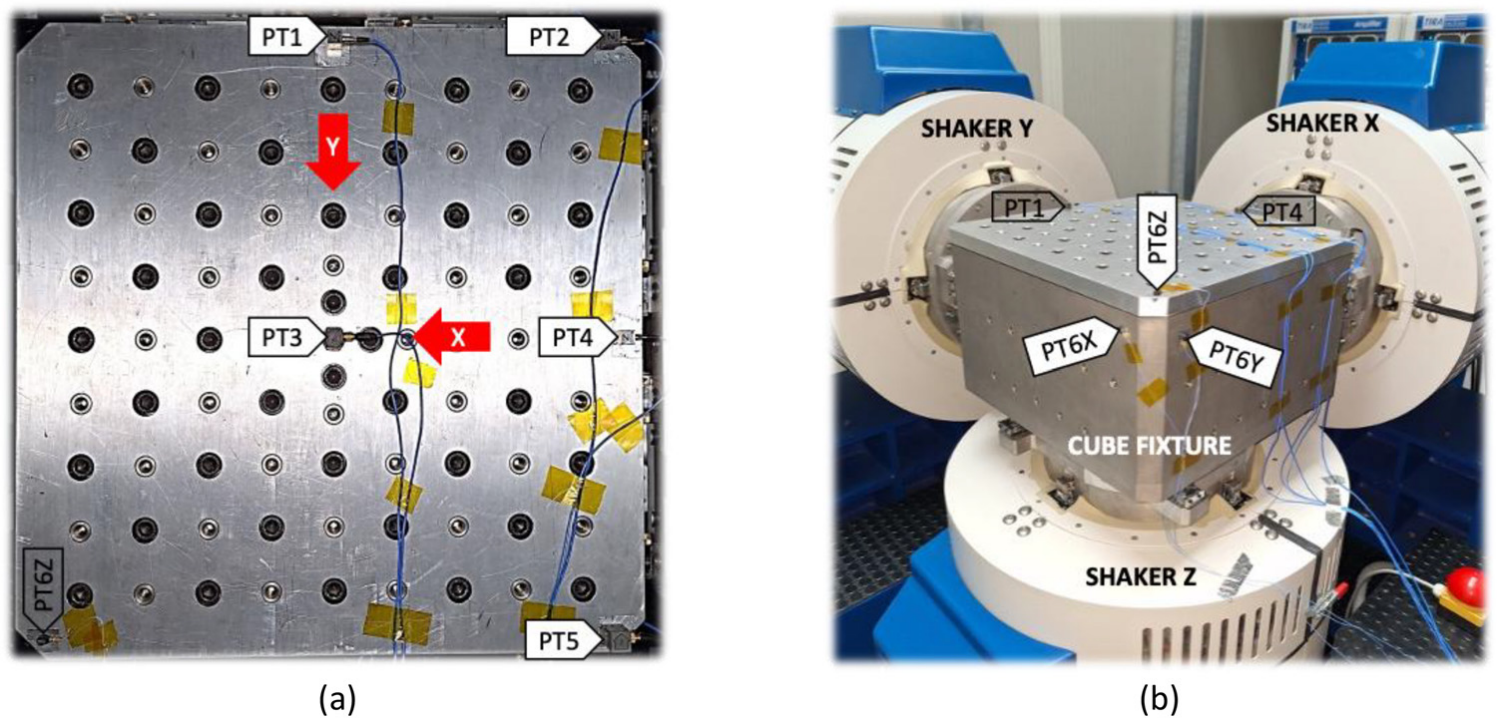
(a) The top view of the cube with the five triaxial accelerometers corresponding to PT1 to 5 and (b) the side view of the cube with the three single-axis accelerometers corresponding to PT6.

A magnesium cube fixture was mounted on the shaker assembly through bolted interfaces and was used as the main experimental fixture investigated in the present dataset. To monitor and control the cube fixture response, five triaxial accelerometers (PT1 to PT5) were mounted on the top surface of the cube (Fig. 6a), and three single-axis accelerometers (PT6X, PT6Y and PT6Z) were mounted on the side surfaces (Fig. 6b). This sensor arrangement enables response measurements in the three directions and supports the different control strategies described in Table 3. The accelerometers were distributed symmetrically over the cube fixture in order to avoid redundant measurement locations and to capture potential bending and torsional responses of the structure. One accelerometer was positioned near the centre of the upper surface, while the others were located close to the fixture edges to improve sensitivity to spatial response variability and structural deformation patterns.

The Jaguar ACP allows the execution of MISO (multi-input single-output) or MIMO (multi-input multi-output) sweep-sine, fixed-frequency sine, and random vibration tests. Each test is parameterised through the Jaguar software (control channels, reference channels, excitation profile, frequency range, levels, and control strategy). Frequency-domain quantities are computed and recorded directly by the ACP during the tests. In random vibration tests, time-history measurements can also be recorded; additional time-domain signals (e.g., strain gauge measurements) may be acquired through external measurement systems when required. The shaker rig can generate simultaneous excitations along the X, Y and Z axes over a frequency range of 5–2000 Hz. A magnesium cube fixture was mounted on the system and used as the fixture interface for the system under test. The main performance specifications of the triaxial shaker rig are reported in Table 1.

**Table 1:** Specifications of the 3D-electrodynamic shaker rig.

Model	EDS-35M0–3
Maximum forces (kN)	35/35/35
Frequency range (Hz)	5–2000
Maximum sinusoidal acceleration (g)	15–10
Maximum random acceleration (g-RMS)	13–10
Maximum displacement pk-pk (mm)	40–35 (30 recommended)
Top surface dimensions of the cube (mm)	500 × 500

All tests remained within the manufacturer-recommended displacement limit of 30 mm pk-pk.

### 4.2 Data acquisition and sensor configuration

For MIMO sweep-sine tests, the Jaguar controller allows the user to specify the relative phase between input excitations. For MIMO random vibration tests, the target excitation is defined through a PSD matrix G(f), with auto-PSDs on the diagonal and cross-PSDs in the off-diagonal terms. These off-diagonal terms are specified by their magnitude, relative phase and coherence. The magnitude-squared coherence $\gamma_{ij}^{2}\left(f\right)$ between control channels i and j is defined as:

(1) $$\gamma_{ij}^{2}\left(f\right)=\frac{|G_{ij}\left(f\right){|}^{2}}{G_{ii}\left(f\right)\;G_{jj}\left(f\right)}$$

where $G_{ii}\left(f\right)$ and $G_{jj}\left(f\right)$ are the auto-PSDs of channels i and j, and $G_{ij}\left(f\right)$ is the complex cross-PSD between these channels, defined as:

(2) $$G_{ij}\left(f\right)=\left|G_{ij}\left(f\right)\right|e^{j\phi_{ij}\left(f\right)}$$

where $\phi_{ij}\left(f\right)$ denotes the phase (file extension .pha) and $|G_{\mathrm{ij}}\left(f\right)|$ the magnitude (file extension .mag). In the released dataset, coherence information is available directly in .coh files, while .mag and .pha files store the quantities used to define the cross-PSD terms.

FRFs are computed with respect to selected reference channels, which correspond to the control channels defined for each test configuration. The FRF estimator and reference-channel selection are those implemented in the Jaguar processing.

The specifications of the eight accelerometers used for the controls and measurements were reported in Table 2.

**Table 2:** Specifications of the eight accelerometers.

Sensor number	Type (PCB Piezotronics)	Serial number	Channel	Sensitivities in mV/g		
				X	Y	Z
PT1	356A32 - Triaxial	LW395297	1 to 3	103.2	97.6	97.8
PT2	356A32 - Triaxial	LW395299	4 to 6	101.1	97.5	98.8
PT3	TLD 356A17	397422	7 to 9	501	505	502
PT4	356A32 - Triaxial	26398	10 to 12	102.2	105.8	104.7
PT5	333A65 - Triaxial	6121	16 to 18	102.6	105.6	105.3
PT6	352A24 - Single-axis	LW501155	13	99.9	-	-
	352A24 - Single-axis	LW501153	14	-	99.3	-
	352A24 - Single-axis	LW501154	15	-	-	102.9

### 4.3 Experimental procedure

Table 3, Table 4 present the control strategies established using triaxial and single-axis accelerometers in order to compare their influence on the dynamic response of the shaker rig under sweep-sine and random vibration excitations. The investigated frequency range was set between 5 and 2000 Hz for all tests. The excitation levels were selected to remain compatible with the shaker capabilities reported in Table 1 and with the dynamic behaviour of the cube fixture used during the tests. The control spectra were defined to ensure stable closed-loop operation and sufficient signal-to-noise ratio over the investigated frequency range.

**Table 3:** Overview of the sweep sine test strategies and associated data recorded.

ID	Strategy	PT	Channel	Axis	Jaguar Run
1	S1: Single-point control	2	4	X	.019.frq .019.frf
			5	Y	
			6	Z	
2	S1: Single-point control	3	7	X	.005.frq .005.frf
			8	Y	
			9	Z	
3	S1: Single-point control	5	16	X	.016.frq .016.frf
			17	Y	
			18	Z	
4	S2: 3 single-axis accelerometers	6	13	X	.008.frq .008.frf
			14	Y	
			15	Z	
5	S3: Distributed-control configuration	4	10	X	.006.frq .006.frf
		1	2	Y	
		3	9	Z

**Table 4:** Overview of the random test strategies and associated data recorded (psd10 and frf10 correspond to the record after 5 min).

ID	Strategy	PT	Channel	Axis	Jaguar Run
6	S1: Single-point control	2	4	X	.073.psd10 .073.frf10 .073.coh10
			5	Y	
			6	Z	
7	S1: Single-point control	3	7	X	.066.psd10 .066.frf10 .066.coh10
			8	Y	
			9	Z	
8	S1: Single-point control	5	16	X	.075.psd10 .075.frf10 .075.coh10
			17	Y	
			18	Z	
9	S2: 3 single-axis accelerometers	6	13	X	.071.psd10 .071.frf10 .071.coh10
			14	Y	
			15	Z	
10	S3: Distributed-control	4	10	X	.069.psd10 .069.frf10 .069.coh10
		1	2	Y	
		3	9	Z	
11	S4: Multi-point rectangular-control	2	4	X	.065.psd10 .065.frf10 .065.coh10
		3	7	X	
		5	16	X	
		3	8	Y	
		6	14	Y	
		5	17	Y	
		2	6	Z	
		3	9	Z	
		6	15	Z	

**Fig. 7:**
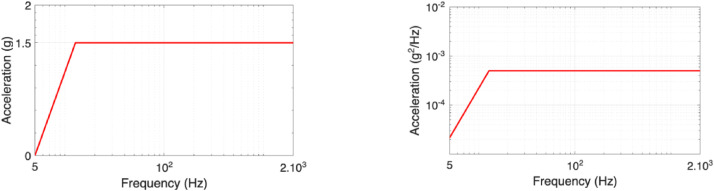
(a) Acceleration spectrum defined for sweep-sine tests (the low-frequency slope corresponds to displacement control) and (b) PSD used for random vibration tests.

Sweep-sine tests were performed at an acceleration level of 1.5 g, with amplitude compression applied above 1500 Hz and a sweep rate of 1 octave/min (Fig. 7a). A displacement-controlled region was introduced at low frequencies to avoid excessive shaker stroke and improve low-frequency control stability, since acceleration control at low frequencies may require large displacement amplitudes.

Random vibration tests were performed using a power spectral density (PSD) corresponding to 1 g RMS applied for 5 min (Fig. 7b). The PSD profile was based on a band-limited white-noise spectrum.

## 5 Supplementary material

This dataset includes complementary acceleration spectra (.FRQ) and Frequency Response Functions (.FRF) for test IDs 1–4 listed in Table 3.

### 5.1 Sweep-sine acceleration dataset

**Fig. 8:**
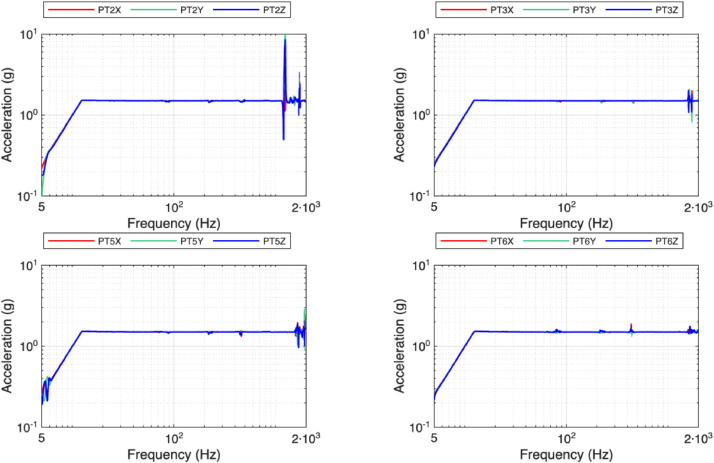
Raw acceleration spectra measured for ID 1 to 4, respectively.

**Fig. 9:**
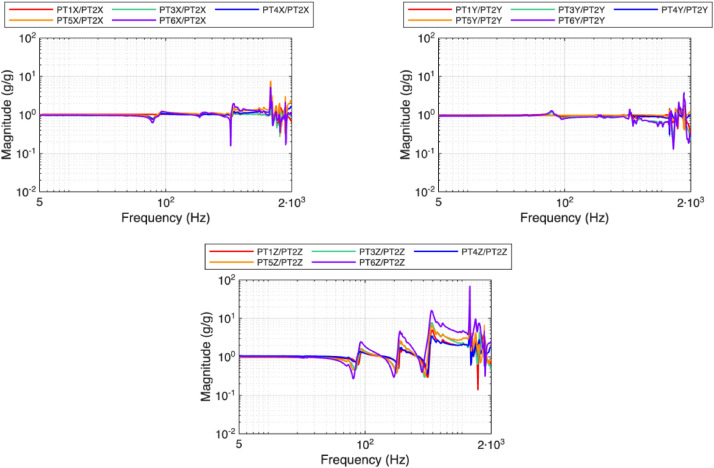
Corresponding FRFs used to compare response measurement locations using S1 with PT2 as control sensor.

Fig. 8 shows the acceleration spectra recorded for ID 1 to 4. Fig. 9 presents the corresponding FRFs used to compare response measurement locations using S1 with PT2 as control sensor. Additional FRFs used to compare response measurement locations for the different control strategies are available in the associated Mendeley Data repository, including configurations using PT3 (S1), PT5 (S1) and PT6 (S2).

### 5.2 Random acceleration dataset

**Fig. 10:**
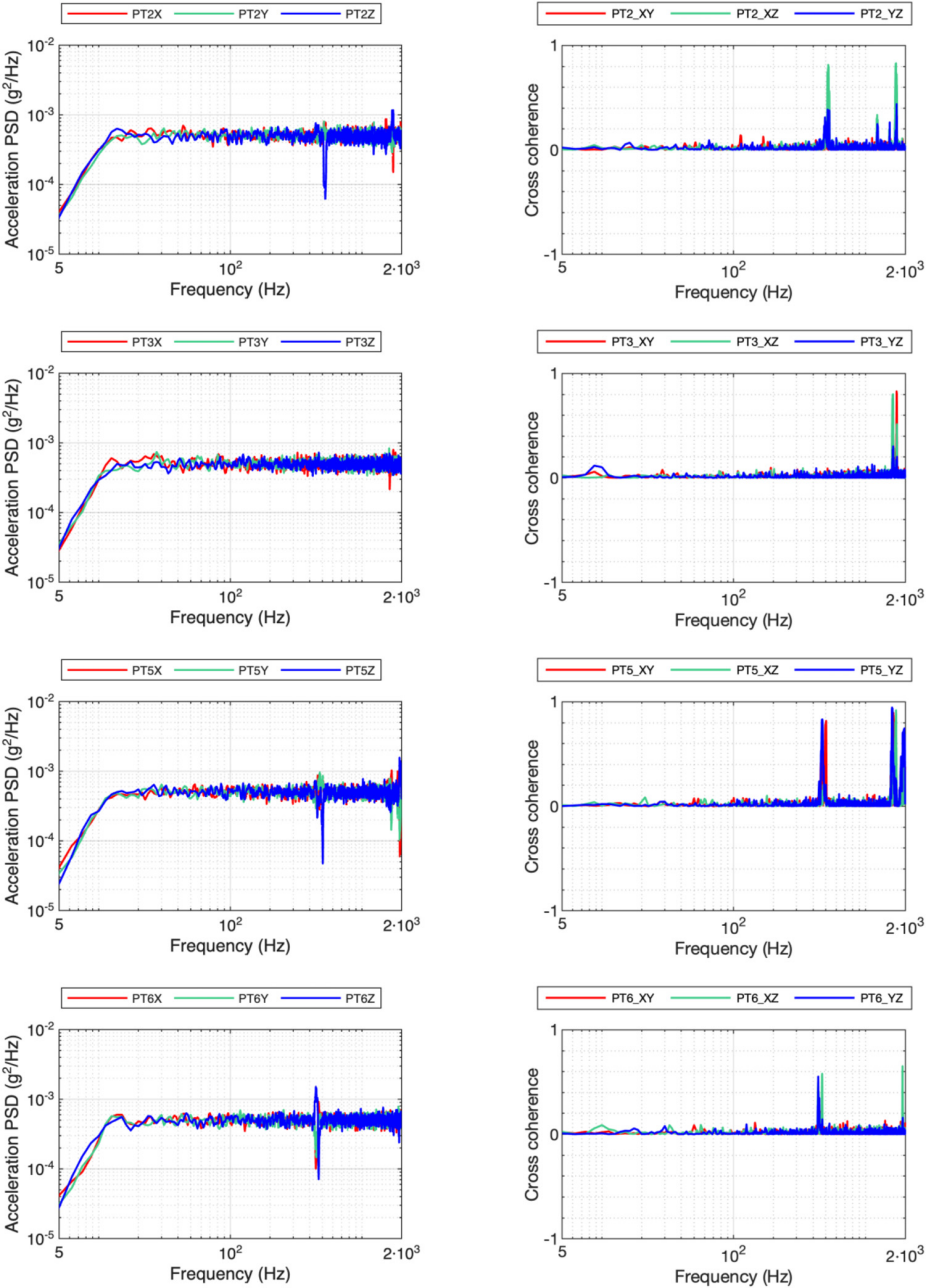
Raw PSDs and cross-coherences for ID 6–9 recorded for the four single-point control configurations.

**Fig. 11:**
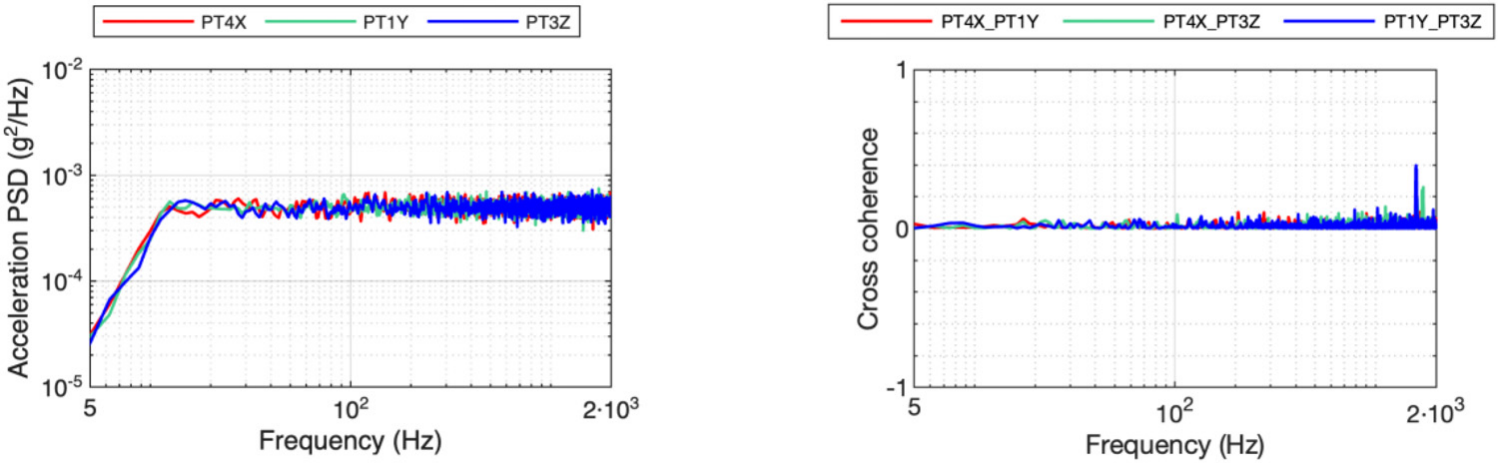
Raw PSDs and cross-coherences recorded for distributed-control configuration on three accelerometers for ID10.

**Fig. 12:**
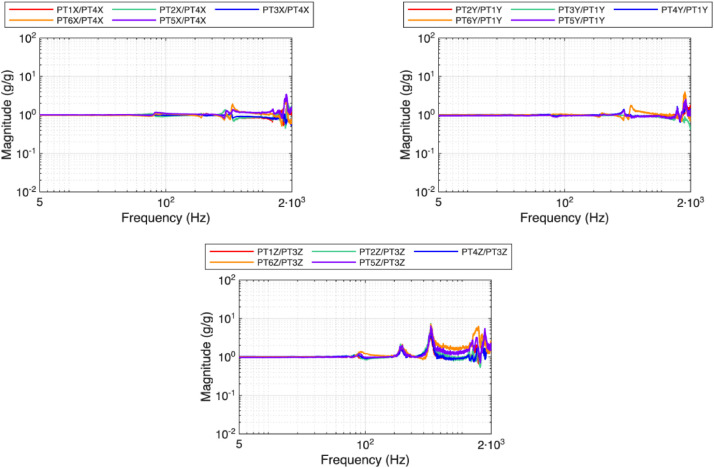
FRFs used to compare response measurement locations with PT4X, PT1Y and PT3Z (S3) under zero coherence.

Figs. 10, 11 show the control PSDs and the associated cross-coherences for tests performed with zero target coherence. Fig. 12 presents the corresponding FRFs used to compare response measurement locations with PT4X, PT1Y and PT3Z (S3) under zero coherence. Additional FRFs used to compare response measurement locations for the different control strategies are available in the associated Mendeley Data repository, including configurations using PT2 (S1), PT3 (S1), PT4 (S1), and PT6 (S2).

## Limitations

The dataset reflects the dynamic behaviour of a triaxial electrodynamic shaker rig equipped with a magnesium cube fixture. Consequently, the identified resonances, spatial variability, and cross-axis coupling characteristics may differ for other fixtures or shaker architectures. Above 1500 Hz, low-amplitude compression was required to maintain control stability, which limited strict proportionality between command and response levels in this frequency range. In addition, the multi-point rectangular-control configuration (S4) could not be fully stabilised because of large spatial response variability. The dataset was acquired without mounted test structures; therefore, the dataset mainly characterises the intrinsic behaviour of the shaker-fixture assembly.

## Ethics Statement

The authors have read and followed the ethical requirements for publication in Data in Brief and confirm that the current work does not involve human subjects, animal experiments, or data collected from social media platforms.

## CRedit Author Statement

**Leila Khalij**: Funding acquisition, Conceptualization, Methodology, Resources, Supervision, Project administration, Validation, Writing - original draft, Writing - review & editing; **Christophe Gautrelet**: Conceptualization, Methodology, Resources, Supervision, Validation, Software, Writing - review & editing; **Carole Dovillaire**: Validation, Writing - review & editing, Technical support for the Jaguar configuration and multiaxial test acquisitions. **Olivier Bareille**: Validation, Writing - review & editing

## Data Availability

Mendeley DataDataset collected using a control strategy on a triaxial platform (Original data).

## References

[1] Habtour E., Connon W., Pohland M.F., Stanton S.C., Paulus M., Dasgupta A. (2014). Review of response and damage of linear and nonlinear systems under multiaxial vibration. Shock Vib.

[2] Aimé M., Banvillet A., Khalij L., Pagnacco E., Chatelet E., Dufour R. (2025). Experimental evaluation of multiaxial test-tailored specifications based on fatigue damage multi-spectra. Int. J. Fatigue.

[3] Hunter N.F., Cross K.R., Nelson G. (2018). Proc. 36th IMAC Conf. Exposition Structural Dynamics.

[4] U.S. Department of Defense, MIL-STD-810H: department of Defense test method standard-environmental engineering considerations and laboratory tests, Method 514.8: Vibration, 2019.

[5] Ao W.K., Tang Q.C., Pavic A. (2024). Decentralised H∞ robust control of MTMDs for mitigating vibration of a slender MDOF floor configuration. Thin-Walled Struct..

[6] Ao W.K., Lu Y., Wang Y.L., Tang Q.C., Pavic A. (2025). Vibration serviceability evaluation on decentralised H∞ robust control of MTMDs of a slender MDOF floor configuration. J. Build. Eng..

[7] Ao W.K., Reynolds P., Caicedo J., Pakzad S. (2017). Dynamics of Civil Structures, Volume 2, Conference Proceedings of the Society for Experimental Mechanics Series.

[8] Liu W., Ni Y.Q., Ao W.K. (2024). Feasibility study of a novel modal decomposition method for low-frequency structure with nonproportionally distributed rate-independent linear damping. Struct. Control Health Monit..

[9] Liu W., Ni Y.Q., Ikago K., Ao W.K. (2023). Seismic control of base-isolated structures using rate-independent damping devices. J. Build. Eng..

[10] Herbut A., Rybak J., Brzakała W. (2020). On a sensor placement methodology for monitoring the vibrations of horizontally excited ground. Sensors.

[11] Underwood M.A., Keller T. (2003). Rectangular control of multi-shaker systems: theory and some practical results. Proc. Inst. Environ. Sci. Technol., ESTECH.

